# The complete mitochondrial genome of *Trachidermus fasciatus* (Scorpaeniformes: Cottidae) and phylogenetic studies of Cottidae

**DOI:** 10.1080/23802359.2018.1445480

**Published:** 2018-02-28

**Authors:** Kehua Zhu, Zhenming Lü, Liqing Liu, Li Gong, Bingjian Liu

**Affiliations:** a National Engineering Laboratory of Marine Germplasm Resources Exploration and Utilization, Zhejiang Ocean University, Zhoushan, China;; b National Engineering Research Center for Facilitated Marine Aquaculture, Marine Science and Technology College, Zhejiang Ocean University, Zhoushan, China

**Keywords:** *Trachidermus fasciatus*, mitogenome, phylogenetic relationship

## Abstract

*Trachidermus fasciatus* is a small catadromous fish and has been listed as a second class state protected aquatic animal since 1988 in China due to the declines in its abundance. We describe the complete mitogenome of *T. fasciatus* in this study. The mitogenome is 16,536 nucleotides long and contains 13 protein-coding genes (PCGs), two ribosomal RNA genes, 22 transfer RNA genes, and two main non-coding regions. The overall base composition includes C (30%), A (26.3%), T (25.5%), and G (18.2%). Moreover, the 13 PCGs encode 3800 amino acids in total, all the PCGs use the initiation codon ATG except COI uses GTG. Most of them have TAA or TAG as the stop codon, except COII, ND4 and Cytb use an incomplete stop codon T. The phylogenetic tree based on the neighbour joining method was constructed to provide relationship within Cottidae, which could be a useful basis for management of this species.

The Roughskin sculpin, *Trachidermus fasciatus* is the only member of the monotypic genus *Trachidermus*. Formerly, they were very abundant along the coast of China, the pollution from rapid urban development, overfishing and the construction of dams and dikes threaten the species so they are now threatened with extinction (Bi et al. 2011). In this study, we determined the complete mitochondrial genome of *T. fasciatus* and explored the phylogenetic relationship based on 12 mitochondrial protein-coding genes (PCGs) located on heavy strand of 18 Cottidae species, contributing to additional information of conservation for this specie (Li et al. 2016).

Sample was collected from Fuchun River in Fuyang, China (27°56′17″N; 119°51′15″E). Specimen was stored in laboratory of Zhejiang Ocean University with accession number 20150826SJL22. Twelve primer pairs were designed to amplify the complete mitogenome. DNA sequences were assembled by Codoncode Aligner. The calculation of base composition and a neighbour joining (NJ) tree were constructed by MEGA5.0 software (Tamura et al. 2011).

The entire genome sequence is 16,536 bp in length (GenBank accession MG784718), consisting of 13 PCGs, 22 tRNA genes, two rRNA genes, one replication origin (OL) and a control region (CR). The overall base composition is 30% A, 26.3% C, 25.5% T, and 18.2% G. Twelve PCGs, 14 tRNA genes, and two rRNA genes were located on the heavy strand, while one PCG (ND6) and eight tRNA genes (tRNA Gln, RNA Ala, tRNA Asn, tRNA Cys, tRNA Tyr, tRNA Ser, tRNA Glu, and tRNA Pro) on the light strand. Thirteen PCGs encode 3800 amino acids in total. All the PCGs use the initiation codon ATG except COI use GTG, which is quite common in other teleost mtDNA (Sergei et al. 2013; Balakirev et al. 2016; Fast et al. 2017). Most of them have TAA or TAG as the stop codon, except COII, ND4 and *Cytb* use an incomplete stop codon T. A total of 52 base pairs in 11 intergenic spacers are found ranging from 1 to 30 bp in length. Eleven overlapping areas (32 bp in total) are observed, notable overlapping occurred at three pairs of PCGs. ATP8 and ATP6 overlapped by 10 nucleotides, ND4L and ND4 by 7 bp, ND5 and ND6 by 4 bp.

The lengths of 12S rRNA located between tRNA^Phe^ and tRNA^Val^ and 16S rRNA located between tRNA^Val^ and tRNA^Leu^ were 945 bp and 1690 bp, respectively (Wang and Xiuying 2008). The origin of light-strand replication (OL) is located in a cluster of five tRNA genes (WANCY) and the CR, with 860 bp, is located between the tRNA-Pro and tRNA-Phe genes (Zhang et al. 2015).

The NJ tree was constructed based on 12 mitochondrial PCGs located on heavy strand of 18 Cottidae species. The results of the present study supports *T. fasciatus* has a closest relationship with Cottus with a bootstrap probability of 99% (Figure 1), which is in accord with the results based on other molecular methods (Yokoyama and Goto 2005).

**Figure 1.**
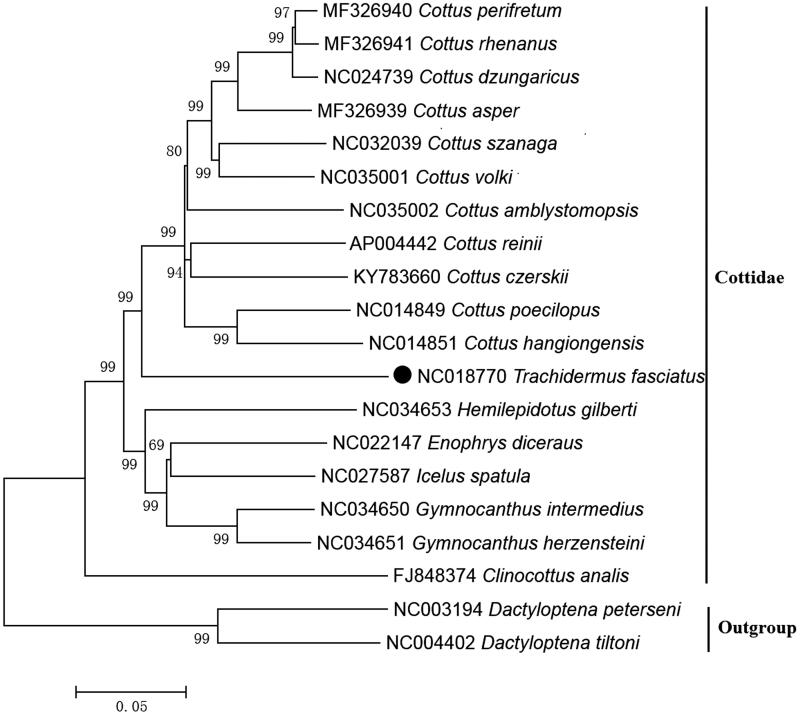
Neighbour joining (NJ) tree of 18 Cottidae species based on 12 PCGs. The bootstrap values are based on 10,000 resamplings. The number at each node is the bootstrap probability. The number before the species name is the GenBank accession number. The genome sequence in this study is labelled with a black spot.
